# 

**Published:** 1868-09

**Authors:** 


### MONTHLY PUBLICATIONS.

ATLANTIC MONTHLY, devoted to Literature, Science, Art and Politics, published by Ticknor & Fields, 124 Tremont Street, Boston, Mass.; office for New York and Brooklyn subscribers, 63 Bleecker Street, New York, a few doors east of Broadway; —$4 a year, single numbers 35 cents.

AMERICAN AGRICULTURIST, $1 50 a year, 41 Park Row, New York, published by Orange Judd & Co., devoted to all that pertains to the farm, garden and orchard.

ARTHUR'S MAGAZINE, $2. a year, 8I1 Chestnut Street, Philadelphia.

BLACKWOOD'S MAGAZINE, or any one of the Reviews, $4; with any one Review, $7. The 4 Reviews, $12. Blackwood and the 4 Reviews, 15.00. 38 Walker Street, west of Broadway. The Leonard Scott Publishing Company, New York.

BEADLE'S MONTHLY; A Magazine of To-Day. $3 a year; 118 William Street, near Fulton. American News Co., Agents, 119 and 121 Nassau Street, New York.

FAMILY TREASURE; A Religious and Literary Monthly, W. T. Findley, D. D., Editor and Publisher. $2. Three copies to one address, $5. 65 W. 4th Street, Cincinnati, Ohio.

GARDENERS' MONTHLY and Horticultural Advertiser, edited by Thomas Meehan, No. 23 North South Street, Philadelphia. $2 a year.

GODEY'S LADIES' BOOK, Vol. 75, edited by Mrs. Sarah J. Hale and L. A. Godey, $3 a year, corner Sixth and Chestnut Streets, Philadelphia, with numerous and beautiful embellishments each month, Fashion plates, Patterns, &c., &c.

HARPER'S WEEKLY, $4 a year, in advance, single copies 10 cents. HARPER'S MAGAZINE, published monthly at Four Dollars a year; postage 24 cents a year; on the WEEKLY, 20 cents. 331 Pearl Street, New York.

HORTICULTURIST, and Journal of Rural Art and Rural Taste, published at $2.50 a year, or 25 cents singly, by Woodward, 37 Park Row, New York. Established in 1846.

HOURS AT HOME, $1.25, published by T. S. Arthur & Co., Philadelphia.

HALF-YEARLY ABSTRACT of The Medical Sciences, being an Analytical and Critical digest of the principal British and Continental Medical works in the preceeding six months, by Henry C. Lea, Philadephia. $2.50 a year, single numbers $1.50

MARYLAND FARMER, devoted to Agriculture, Horticulture, Rural Economy and Mechanic Arts, published by S Sands, Mills & Co. at $1.50 a year. 21 South Calbert Street, Baltimore, Md.

MERRY'S MUSEUM, $1.50 a year, 172 William Street, New York.

MOTHER'S JOURNAL and Family Visitant, edited by Miss Caroline O. Hiscox and published by Sheldon & Company, 500 Broadway, New York.

NEW ENGLAND FARMER, published by Eaton & Co., 34 Merchants Row, Boston. $1.50 a year, single copies 15 cents; 52 pages, 8vo, monthly.

SABBATH AT HOME, an illustrated religious magazine for the family, published by the American Tract Society, 28 Cornhill, Boston, and at 13 Bible House, New York, by the Messrs. Broughton & Wyman. $2 a year, or $2 50.for six months.

PACKARD'S MONTHLY, New-York. $1 a year.

